# Suppression of the immune system as a critical step for bone formation from allogeneic osteoprogenitors implanted in rats

**DOI:** 10.1111/jcmm.12172

**Published:** 2013-11-17

**Authors:** Anindita Chatterjea, Vanessa LS LaPointe, Jacqueline Alblas, Supriyo Chatterjea, Clemens A Blitterswijk, Jan Boer

**Affiliations:** a Department of Tissue Regeneration MIRA Institute for Biomedical Technology and Technical Medicine, University of TwenteEnschede, The Netherlands; b Department of Orthopaedics, University Medical Centre UtrechtUtrecht, The Netherlands; c Pervasive Systems Group, University of TwenteEnschede, The Netherlands

**Keywords:** Mesenchymal stromal cells, bone tissue engineering, allogeneic cells, immunology, immunosuppression

## Abstract

The surface marker profile of mesenchymal stromal cells (MSCs) suggests that they can escape detection by the immune system of an allogeneic host. This could be an optimal strategy for bone regeneration applications, where off-the-shelf cells could be implanted to heal bone defects. However, it is unknown how pre-differentiation of MSCs to an osteogenic lineage, a means of improving bone formation, affects their immunogenicity. Using immunohistological techniques in a rat ectopic implantation model, we demonstrate that allogeneic osteoprogenitors mount a T cell- and B cell-mediated immune response resulting in an absence of *in vivo* bone formation. Suppression of the host immune response with daily administration of an immunosuppressant, FK506, is effective in preventing the immune attack on the allogeneic osteoprogenitors. In the immunosuppressed environment, the allogeneic osteoprogenitors are capable of generating bone in amounts similar to those of syngeneic cells. However, using osteoprogenitors from one of the allogeneic donors led to newly deposited bone that was attacked by the host immune system, despite the continued administration of the immunosuppressant. This suggests that, although using an immunosuppressant can potentially suppress the immune attack on the allogeneic cells, optimizing the dose of the immunosuppressant may be crucial to ensure bone formation within the allogeneic environment. Overall, allografts comprising osteoprogenitors derived from allogeneic MSCs have the potential to be used in bone regeneration applications.

## Introduction

Tissue engineered constructs generated with bone marrow-derived mesenchymal stromal cells (MSCs) seeded on scaffolds represent a possible alternative to autologous bone grafts for the treatment of bone defects. Barriers to the clinical usefulness of patient-derived MSCs include the large inter-donor variability 1–2 and the increasing evidence that a pre-differentiation protocol prior to implantation may be necessary for optimal bone formation 3–4. Recent advances have determined a selection of *in vitro* markers that may help predict *in vivo* performance 2,5, but the best test of a donor-derived cell population remains *in vivo* implantation, a time-consuming and costly process. One way to overcome these limitations would be to use allogeneic MSCs, which could be selected, tested, and pre-differentiated prior to implantation, thereby improving the clinical outcome for patients undergoing a bone graft procedure.

While there have been reports of allogeneic MSCs employed to heal bone defects, there remain concerns on the immune response they may elicit upon transplantation and how this affects their ability to contribute to tissue formation 7. Mesenchymal stromal cells are generally thought to express low levels of MHC class I, to lack MHC class II, and to not express the co-stimulatory molecules required to stimulate the T cell response 8. They have also been shown to suppress lymphocyte alloreactivity *in vivo*, by directly inhibiting T cell proliferation and secretion of T_H_1 lymphokines, such as IFN-γ 9,10. Rat MSCs have been shown to express RT1A (MHC class I), but not RT1B (MHC class II) 12,13. Taken together, these data suggest that MSCs are poorly immunogenic and can be transplanted in an allogeneic host without rejection or the need for additional immunosuppressive therapy 15. Generally, *in vivo* experiments support this immunoprivileged status, with successful implantation of allogeneic MSCs in many systems, and species including goats 16–17, pigs 18–19 and dogs 20. However, there have also been reports of unsuccessful allogeneic transplants, including in rodents, where allogeneic MSCs transplanted along with hydroxyapatite scaffolds failed to form bone 21. Furthermore, only a few studies have determined how a pre-differentiation protocol may affect immunogenicity, despite evidence that this may be critical. For example, when rabbit MSCs underwent osteogenic pre-differentiation, they were still immunoprivileged and immunomodulatory, but the latter was lost upon transplantation 22. Finally, very little is known about the mechanism by which osteoprogenitors derived from allogeneic MSCs may interact with the cells of the immune system and how this correlates with bone formation.

The purpose of this study is to determine whether allogeneic MSCs that have been pre-differentiated to the osteogenic lineage can escape detection by the immune system and induce bone formation. A rat ectopic implantation model was chosen, in which we monitored the activation of T cells and B cells in response to the implantation of a ceramic combined with syngeneic and allogeneic osteoprogenitors. While the allogeneic osteoprogenitors were capable of forming bone in a nude mouse, implantation in an immunocompetent rat led to a T cell- and B cell-mediated immune response that resulted in an abolition of bone formation. We then administered FK506, an immunosuppressant widely used in organ transplantations that inhibits the secretion of T cell-derived soluble mediators such as IL-2 and -4 as well as IFN-γ. These cytokines are required for the maturation of B cells and, as only mature B cells can present antigens to T cells to activate them, FK506 can be considered to block the activation of both T and B cells 23. Administration of the immunosuppressant was able to partially rescue bone formation, and therefore represents a potential strategy for the use of allogeneic osteoprogenitor transplants.

## Materials and methods

### Cell isolation and culture

Mesenchymal stromal cells were obtained from the femoral shafts of two 6-week-old male inbred Fischer 344 (F344/NCrHsd, Harlan Laboratories, Boxmeer, The Netherlands) or outbred Wistar (HsdOla: WI, Harlan Laboratories) rats. Cells originating from each of two donor rats of each strain are hereafter referred to as F1 and F2 for those isolated from the Fischer 344 rats, and W1 and W2 for those isolated from the Wistar rats. To isolate MSCs, both ends of the rat femurs were cut at the epiphysis and the bone marrow was flushed out with 10 ml of culture medium expelled from a syringe through a 20-gauge needle according to a previously described protocol 24. The bone marrow from each femur was plated in two 75 cm^2^ flasks and cultured in proliferation medium (basic medium supplemented with 1 ng/ml basic fibroblast growth factor (Instruchemie, Delfzijl, The Netherlands)). Basic medium consisted of minimal essential medium alpha (αMEM; Gibco, Bleiswijk, The Netherlands) supplemented with 15% (v/v) foetal bovine serum (FBS; Lonza, Breda, The Netherlands), 0.2 mM ascorbic acid (Sigma-Aldrich, Zwijndrecht, The Netherlands), 2 mM l-glutamine (Gibco), 100 U/ml penicillin (Gibco) and 100 μg/ml streptomycin (Gibco). Cultures were maintained in a humidified atmosphere at 37°C with 5% CO_2_ and medium was changed twice weekly. Cells were purified by their adherence to tissue culture plastic during subsequent medium changes. Subconfluent MSCs were trypsinized and either further subcultured or cryopreserved for future use. Their ability to undergo osteogenic and adipogenic differentiation was verified using Alizarin Red S and Oil Red O staining, according to standard differentiation protocols.

### Scaffold fabrication

Biphasic calcium phosphate (BCP) ceramics were kindly provided by Dr Huipin Yuan, University of Twente, The Netherlands. Briefly, the BCP composed of 20% tricalcium phosphate and 80% hydroxyapatite was produced according to the H_2_O_2_ method, including naphthalene, as described previously 25. The material was sintered at 1300°C to produce an average granule size of 2–3 mm, a specific surface area of 0.2 m^2^/g, a microporosity (volume percentage of micropores <10 μm) of 8.7%, and a calcium release of 4.2 ± 0.4 ppm 26.

### Validation of bone-forming capacity in nude mice

All *in vivo* experiments were approved by the local animal experimental committee. To validate the bone-forming capacity of the allogeneic and syngeneic MSCs, they were first differentiated into osteoprogenitors by culture in osteogenic medium [basic medium supplemented with 0.1 μM dexamethasone (Sigma-Aldrich)] for 5 days. A total of 600,000 cells were implanted with three BCP ceramic particles in the subcutaneous pockets on the dorsum of six nude mice (Hsd-cpb: NMRI-nu, Harlan Laboratories) for 6 weeks. Mice were anaesthetized by inhalation of isoflurane and the incisions were closed using a vicryl 5-0 suture. They were killed with carbon dioxide and constructs were explanted and prepared for histology.

### Generation of the syngeneic and allogeneic constructs

Isografts were generated with the Fischer-derived cells (syngeneic donors) and allografts were generated with the Wistar-derived cells (allogeneic donors). In all cases, MSCs were pre-differentiated to osteoprogenitors, and 600,000 cells (passage 3) were gently dispersed over BCP particles. After 4 hours of incubation, 2 ml of culture medium was slowly added and the cell-scaffold constructs were cultured for a total of 5 days in a humidified atmosphere at 37°C with 5% CO_2_ prior to implantation in 6-week-old Fischer 344 rats (with the same protocol as implantation in nude mice). As a control, all rats were also implanted with a BCP ceramic construct lacking cells.

### Characterization of the immune response

To determine the immune response of Fisher rats to syngeneic and allogeneic osteoprogenitor-based BCP constructs, implants were stained with antibodies against T cells (CD3) or B cells (*pan*). The constructs (from *N* = 6 recipient rats in each group) were retrieved after 12 days *in vivo,* fixed in 4% (w/v) paraformaldehyde, embedded in agarose, decalcified with 12.5% (w/v) ethylenediaminetetraacetic acid, and dehydrated with sequential ethanol series. They were then incubated overnight in butanol, in a butanol and paraffin (50:50 v/v) solution for 8 hrs, and were embedded in paraffin. A microtome was used to cut 3-μm-thick sections. Antigen retrieval (1:50 dilution of the Target Retrieval Solution, pH 9, EnVisionTMFlex; Dako, Cambridgeshire, UK) was performed according to the manufacturer’s instructions on sections to be stained for T cells, and all sections were blocked for endogenous peroxidase activity with hydrogen peroxide for 5 min. Primary antibodies were polyclonal rabbit anti-CD3 (1:200; Dako) to stain T cells and monoclonal mouse anti-B cell (1:50, clone Ki-B1R; Acris; Bio-Connect, Huissen, The Netherlands), and were diluted in PBS and incubated for 20 min. Antibodies were visualized using an EnVisionTMFlex mini kit (Dako), which is based on DAB, according to the manufacturer’s instructions. The anti-B cell antibody signal was amplified with the EnVision FLEX+ Mouse (LINKER) from Dako. Finally, haematoxylin was used to counterstain the nuclei. Positive controls for the staining on sections of rat spleen are available in the Supplementary Data (Fig. S1). The slides were visualized on a bright-field microscope (E600; Nikon, Amsterdam, The Netherlands).

### Histology and histomorphometry of the explanted samples

To visualize and quantify bone formation, constructs were retrieved after 6 weeks *in vivo*, fixed in 4% (w/v) paraformaldehyde, dehydrated in an industrial microwave with JFC dehydration solution (Leica Microsystems, Rijswijk, The Netherlands), and transferred into a methyl methacrylate solution (L.T.I, Bilthoven, The Netherlands) to polymerize at 37°C for 3 days. Embedded constructs were sectioned to 10–15 μm thickness with a modified interlocked diamond saw (Leica Microtome, Nussloch, Germany). They were etched with HCl (0.75% (v/v) HCl in ethanol for 1 min) and stained for 1 min in 1% (w/v in 0.1 M sodium borate, pH 8.5) methylene blue and 0.3% (w/v in water) basic fuchsin. Sections were visualized (Nikon E600) and scored for bone formation. Three non-consecutive sections per sample per animal were digitally scanned and quantified with Adobe Photoshop (CS5 Extended, Adobe, Amsterdam, The Netherlands) as follows: bone and scaffold areas were differentially pseudo-coloured and the ratio between pixels of each colour was converted to percentage of bone area per scaffold area. Statistical analysis was performed with Student’s paired *t*-test or with one-way anova followed by Tukey’s multiple comparison test, with *P* < 0.05 considered statistically significant.

### Immunosuppression in immunocompetent Fisher rats

To uncover the role of the immune response in bone formation, one group of rats received an immunosuppressant, FK506 (Tacrolimus; Astellas Pharma, Leiderdorp, The Netherlands), at a dosage of 1 mg/kg daily intramuscularly, while the control group received a saline injection. This dosage was based on organ transplantation studies in rats showing a positive therapeutic outcome with no side effects 27–28. Rats were anaesthetized by inhalation of isoflurane and the incisions were closed with a vicryl 5-0 suture. Killing with carbon dioxide occurred either after 12 days or 6 weeks to determine the immune response and bone formation respectively. Following explantation, constructs were prepared for histology as described above.

### Dynamics of bone formation

To compare the dynamics of bone apposition between the constructs composed of syngeneic and allogeneic osteoprogenitors, isografts and allografts were implanted subcutaneously in 12 immunocompetent Fischer 344 rats, with the same implantation protocol as in the other experiments. Sequential fluorochrome labels were infused at 2 weeks [calcein green in 2% (w/v) NaHCO_3_, 10 mg/kg subcutaneously, Sigma-Aldrich] and 4 weeks [xylenol orange in 1% (w/v) NaHCO_3_, 100 mg/kg intravenously, Sigma-Aldrich] 29. Immunosuppression was induced in six of the 12 rats with FK506 at a dosage of 1 mg/kg daily intramuscularly for 6 weeks. The other six rats received saline injections. All rats were killed after 6 weeks and constructs were explanted to visualize bone formation on a fluorescent microscope (Nikon E600) equipped with a quadruple filter block (XF57, dichroic mirror 400, 485, 558 and 640 nm, Omega Filters, Didam, The Netherlands).

## Results

### Rat MSCs are multipotent and can form bone *in vivo*

**Figure 1:**
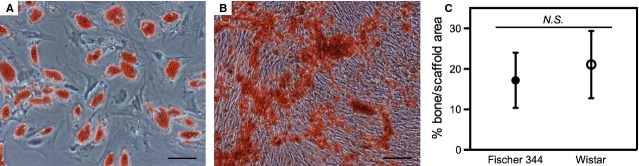
Rat mesenchymal stromal cells (MSCs) are multipotent and can form bone *in vivo*. Multilineage differentiation capacity was assessed by subjecting MSCs to adipogenic and osteogenic differentiation protocols and staining for lipids (A) and mineralization (B) respectively (scale: 50 μm). Bone formation capacity was assessed by implanting osteoprogenitors in nude mice, followed by a quantification of bone area/scaffold area (mean ± SD) in histological sections (C), where no significant difference (*N.S*.) in bone-forming capacity was found in cells derived from either Fischer 344 or Wistar rats.

To demonstrate that MSCs isolated from the bone marrow of two inbred Fischer 344 (donors F1 and F2) and two outbred Wistar (donors W1 and W2) rats were multipotent, they were differentiated towards two mesenchymal lineages. Figures 1A and B are representative images of adipogenic and osteogenic differentiation, respectively. To demonstrate that these cells could form bone *in vivo*, they were pre-differentiated to osteoprogenitors and implanted ectopically in nude mice for 6 weeks along with the BCP ceramic particles. Importantly, there were no statistically significant differences in bone formation comparing cells derived from the Fisher 344 and Wistar rats (17.2 ± 6.8 and 21.1 ± 8.3% bone area/scaffold area respectively; Fig. 1C).

### Empty ceramics do not induce a T cell- and B cell-mediated immune response

**Figure 2:**
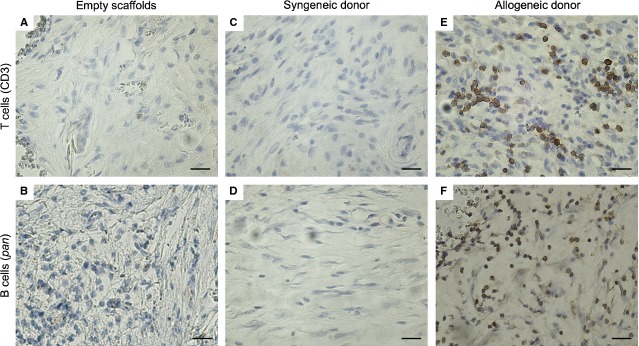
Allogeneic osteoprogenitors induce a T cell and B cell immune response when implanted with biphasic calcium phosphate particles *in vivo*. After 12 days, immunohistochemistry was used to identify T cells (positive for CD3) and B cells (both visualized with DAB; brown). Ceramics implanted without cells (A and B) and with syngeneic osteoprogenitors (C and D) did not recruit T cells or B cells, in contrast to those implanted with allogeneic osteoprogenitors (E and F). Nuclei were counterstained with haematoxylin (blue). Each image is representative of explants from *N* = 6 rats. Scale: 50 μm.

To determine the immune response elicited by the BCP ceramics alone, the ceramics without cells were cultured in osteogenic medium for 5 days and then implanted subcutaneously in two randomly selected immunosuppressed (with FK506) and non-immunosuppressed (without FK506) Fischer 344 rats. The ceramics alone did not initiate a T cell- or B cell-mediated immune response in either the immunosuppressed (data not shown) or non-immunosuppressed (Fig. 2A and B) rats.

### Allogeneic osteoprogenitors elicit an immune response upon implantation

To investigate the immunogenicity of allogeneic constructs, osteoprogenitors from Fischer 344 and Wistar rats were implanted with BCP scaffolds in ectopic locations in immunocompetent Fischer 344 rats for 12 days, after which they were explanted, paraffin embedded, and sectioned. The sections were stained with an anti-CD3 antibody, which recognizes its epitope on all mature T cells that play a role in cell-mediated immunity or a *pan*-B cell antibody, which is present on all B cells and plasma cells, the mediators of humoural immunity. While the constructs seeded with syngeneic cells did not stain positive for either the CD3 antibody (Fig. 2C) or the B cell antibody (Fig. 2D), the allogeneic constructs showed a strongly positive staining for CD3 (Fig. 2E) and B cells (Fig. 2F). This indicated that the allogeneic osteoprogenitors are recognized as foreign by the host immune system, resulting in a T cell- and B cell-mediated immune response, while the syngeneic osteoprogenitors evade immune detection.

### Immune response is associated with the absence of bone formation *in vivo*

**Figure 3:**
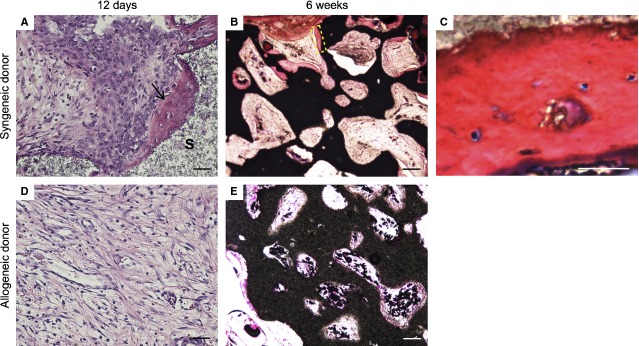
Syngeneic, but not allogeneic, osteoprogenitors form bone when implanted with biphasic calcium phosphate particles *in vivo*. Immunohistochemistry revealed bone formation when rats were implanted with syngeneic cells (A–C). Haematoxylin and eosin (A) staining demonstrated immature bone formation (arrow: bone, S: scaffold) as early as 12 days and methylene blue and basic fuchsin (B) demonstrated mature bone by 6 weeks (C: high magnification of dashed rectangle in B). In contrast, rats implanted with allogeneic osteoprogenitors displayed no bone formation (D and E). Scales in A and D: 50 μm, B and E: 200 μm, C: 10 μm. Each image is representative of explants from *N* = 6 rats.

To determine the consequences of the differential immune response to syngeneic and allogeneic osteoprogenitors, bone formation was assessed after implantation. After only 12 days, four of six implants demonstrated early signs of bone formation (Fig. 3A) and all syngeneic constructs generated bone after 6 weeks (Fig. 3B and C). In contrast, no bone formation was observed in rats implanted with an allogeneic construct, even after 6 weeks (Fig. 3D and E). This formed the basis of our hypothesis that suppressing the immune response could rescue the bone-forming capacity of allogeneic cells.

### Administration of immunosuppressant effectively blocks the T and B cell recruitment

**Figure 4:**
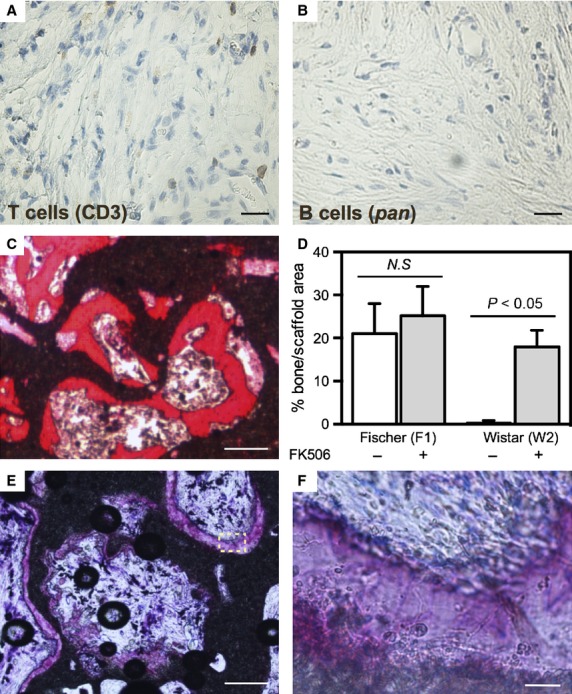
Inhibiting the immune response permits bone formation in allogeneic constructs. Following administration of FK506, the T cell and B cell response to allogeneic osteoprogenitors was suppressed (A and B, scale: 50 μm) and bone formation was observed (C: methylene blue and basic fuchsin, scale: 100 μm) with cells from donor W2. Quantification of bone area/scaffold area demonstrates that FK506 had no significant (*N.S*.) effect on bone formation in isografts, but enabled bone formation in allografts (D). Allogeneic cells from a second donor (W1) elicited a late immune response, which impaired bone formation (E, methylene blue and basic fuchsin, scale: 100 μm, F: zoomed area of E, scale: 5 μm). Each image is representative of explants from *N* = 6 rats.

Given the correlation between bone formation and a T cell- and B cell-mediated immune response, we next sought to determine whether suppressing the immune response could enable allogeneic osteoprogenitors to form bone. FK506, an immunosuppressant, was administered daily to the recipient rats. After 12 days of *in vivo* implantation, the constructs generated from syngeneic and allogeneic osteoprogenitors were explanted and analysed for the presence of an immune response. In agreement with the previous experiment (without FK506 administration), there were no T cells or B cells in the isografts with syngeneic cells (data not shown). However, with the supplementation of the immunosuppressant, there were also no T cells or B cells in the allografts with allogeneic cells (Fig. 4A and B). This validated that the FK506 effectively eliminated the immune response associated with the allogeneic cells.

### Allogeneic osteoprogenitors can generate bone within an immunosuppressed milieu

We next sought to determine if the suppression of the cellular and adaptive immunity was a possible intervention to permit the use of allogeneic osteoprogenitors for bone regeneration purposes. FK506 was administered daily for 6 weeks to the rats receiving the constructs generated using the syngeneic as well as the allogeneic osteoprogenitors. The isografts seeded with the syngeneic cells from both donors generated new bone regardless of administration of FK506 and, importantly, quantification of bone formation determined a statistically similar (*P* > 0.05) amount of bone in the presence or absence of FK506 (24% and 21% of bone area/scaffold area respectively), indicating that the drug had no direct effect on bone formation (Fig. 4D). Interestingly, the administration of an immunosuppressant proved to be a successful strategy to permit bone formation in the allografts generated from allogeneic donor W2 osteoprogenitors. Although no bone was generated in the absence of FK506 (Fig. 3D–F), an average of 17.9% bone was measured when FK506 was administered (Fig. 4C and D). Indeed, with immunosuppression, there was no statistically significant difference in bone formation between the syngeneic donor and allogeneic donor W2 (Fig. 4D). However, this result was not reproduced with osteoprogenitors from a second allogeneic donor (W1). In that case, bone deposition was observed in close contact with the ceramic pores (Fig. 4E and F), but, while the location and gross appearance of the deposit was suggestive of bone, closer histological examination under higher magnification revealed the absence of bone lining cells, osteoblasts and osteoclasts (Fig. 4F). Here, despite the administration of FK506, it appeared that newly formed bone was attacked, resulting in the destruction of the cellular component, and subsequent generation of a cell-free mineralized matrix.

### Dynamics of bone deposition in immunosuppressed rats

**Figure 5:**
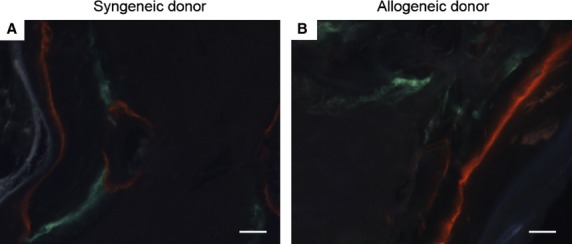
No difference in bone formation dynamics was observed in immunosuppressed rats. To uncover any difference in the dynamics of bone formation, two fluorescent dyes were administered to immunosuppressed rats (calcein green at 2 weeks and xylenol orange at 4 weeks). Upon explantation after 6 weeks, no significant differences were noted in the bone formation dynamics comparing syngeneic osteoprogenitors (A) and allogeneic osteoprogenitors (B), suggesting that, in both cases, bone formation commenced before 2 weeks and continued beyond 4 weeks. Scale: 100 μm. Each image is representative of explants from *N* = 6 rats.

To determine if the initiation and progression of bone deposition varied between the constructs composed of syngeneic and allogeneic osteoprogenitors, a fluorochrome study was performed. Rats were implanted with isografts (syngeneic donor F2) and allografts (allogeneic donor W2) and given daily injections of FK506 for the 6-week duration. Calcein green was administered as a single dose after 2 weeks of implantation, while xylenol orange was administered as a single dose at 4 weeks. The resulting micrographs demonstrated that, in both syngeneic (Fig. 5A) and allogeneic (Fig. 5B) constructs, bone formation started before 2 weeks and continued even after 4 weeks. This indicated that the dynamics of bone deposition were unaffected by the donor cell source.

## Discussion

Mesenchymal stromal cells are generally considered non-immunogenic; however, there is a concern with using allogeneic MSCs for bone tissue regeneration applications, because of the potential loss of their immunoprivileged status upon commitment to the osteogenic lineage. This is an especially important consideration in bone tissue engineering, where a pre-osteogenic commitment protocol can be necessary to prime MSCs for bone formation. In this study, we found that osteoprogenitors derived from allogeneic rat MSCs elicit a host T cell- and B cell-mediated immune response, resulting in an absence of *in vivo* bone formation. We also demonstrated the potential to rescue bone formation by administration of an immunomodulatory drug.

The detrimental outcome associated with allogeneic MSC transplants has been previously demonstrated in the case of bone formation 21. Indeed, allogeneic MSCs have been combined with hydroxyapatite discs and, when implanted in rats, they failed to form bone 28–30. Similar to our findings, the authors of both studies determined that administration of an immunosuppressant could enable bone formation. However, they did not directly establish the role of the immune response, nor did they use a pre-differentiation protocol, instead opting to implant naïve MSCs.

Our study supports the notion that rat MSCs may lose some of their immunoprivileged status upon differentiation. The same outcome found in this study may not necessarily translate to other animal models or organs other than bone. While some studies have found that the immunoprivileged status of MSCs is retained even after their differentiation 31, others report an increase in MHC expression following differentiation 32. In the latter study, allogeneic cells implanted directly into an infarcted cardiac muscle were cleared within 5 weeks, while syngeneic cells were not. Whether this was because of the acquisition of a non-immunoprivileged phenotype upon differentiation, or whether these MSCs were immunogenic in their naïve state is unknown. In an example involving bone formation, allogeneic MSCs differentiated until they were mineralized *in vitro* were implanted with hydroxyapatite, after which they failed to form bone in immunocompetent rats 33. This was attributed to the immune response when bone formation was observed in isografts, although no T cell or B cell response was directly observed.

We further aimed to see whether an immunosuppressive therapy prescribed in organ transplantation could be used to prevent a host immune response to allogeneic cells. FK506, a commonly used immunosuppressant, successfully inhibited T cell and B cell recruitment and rescued bone formation in one allogeneic donor. Using cells from the other donor, bone formed, but histological analysis indicated that it was attacked at a later time-point. It is unsurprising that some allogeneic donors elicit a stronger immune response than others, and this could necessitate a higher dose or polytherapy with other immunosuppressants. We administrated FK506 daily in this study, which is in agreement with a previous report, where intermittent administration of the immunosuppressant in conjunction with an allogeneic whole bone marrow transplant resulted in bone with empty lacunae surrounded by cellular infiltration, suggestive of an immune attack following the discontinuation of the immunosuppressant after 2 weeks 28. Thus, it may be interesting to attempt future studies to test the ideal combination of doses and durations of immunosuppressants to protect the implanted allogeneic cells for a window period during which they can initiate the bone formation of the host cells *via* paracrine effects. This may provide an attractive possibility of avoiding a lifelong dependency on immunosuppressants for clinical cases involving allogeneic MSCs for bone formation.

## Conclusion

The effects of pre-committing MSCs to the osteogenic lineage on their immunoprivileged status and bone-forming capacity *in vivo* were previously unknown. This study demonstrated that rat osteoprogenitors derived from MSCs in an allogeneic environment are not intrinsically immunoprivileged or immunosuppressive. However, under appropriate immunosuppressant therapy, they can survive and generate bone *in vivo*. Therefore, allografts composed of allogeneic osteoprogenitors and ceramics represent a potential approach to bone regeneration.

## Supporting information

Figure S1 Spleen sections stained with primary antibodies against B cells (A) and T cells (B).

Figure S2 Inhibiting the immune response does not affect bone formation in syngeneic constructs. Following administration of FK506, bone formation was observed (A: methylene blue and basic fuchsin, scale: 100 lm) in donor W2, similar to the amount of bone seen without immunosuppression. B: high magnification (scale: 5 μm).
