# Supplementary figures and images for: Suppression of the immune system as a critical step for bone formation from allogeneic osteoprogenitors implanted in rats
Source: J Cell Mol Med. 2013 Nov 17;18(1):134–42. doi: 10.1111/jcmm.12172 (PMC3916125; doi:10.1111/jcmm.12172)

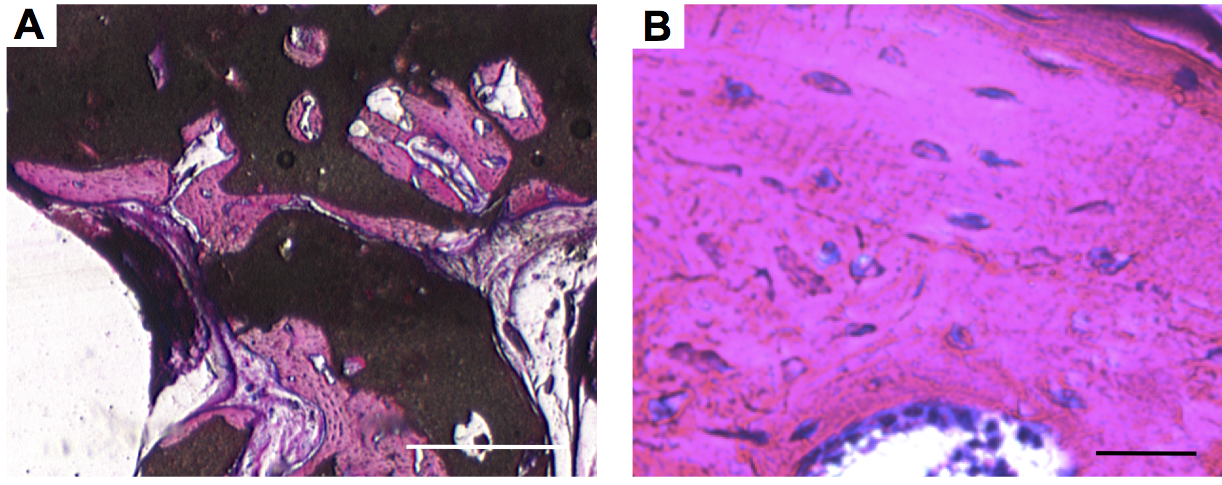

Supplement: Figure S1 — Spleen sections stained with primary antibodies against B cells (A) and T cells (B). [file jcmm0018-0134-sd1.tiff]

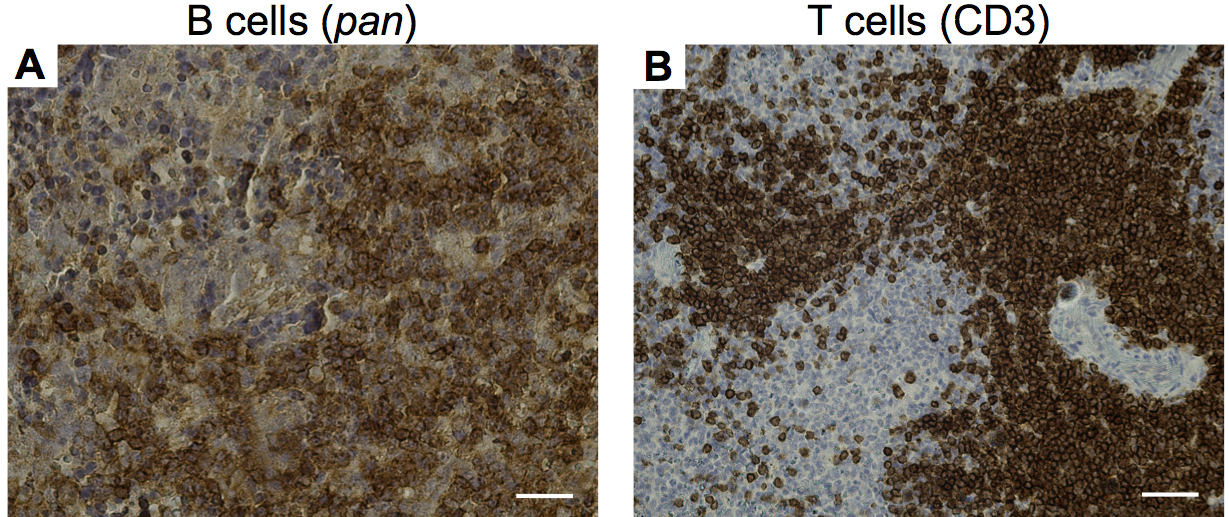

Supplement: Figure S2 — Inhibiting the immune response does not affect bone formation in syngeneic constructs. Following administration of FK506, bone formation was observed (A: methylene blue and basic fuchsin, scale: 100 lm) in donor W2, similar to the amount of bone seen without immunosuppression. B: high magnification (scale: 5 μm). [file jcmm0018-0134-sd2.tiff]
